# Kinetics of Respiratory Syncytial Virus (RSV) Memphis Strain 37 (M37) Infection in the Respiratory Tract of Newborn Lambs as an RSV Infection Model for Human Infants

**DOI:** 10.1371/journal.pone.0143580

**Published:** 2015-12-07

**Authors:** Alejandro Larios Mora, Laurent Detalle, Albert Van Geelen, Michael S. Davis, Thomas Stohr, Jack M. Gallup, Mark R. Ackermann

**Affiliations:** 1 Department of Veterinary Pathology, Iowa State University, Ames, Iowa, United States of America; 2 Ablynx NV, Zwijnaarde, Belgium; 3 Center for Veterinary Health Sciences, Oklahoma State University, Stillwater, Oklahoma, United States of America; National Heart and Lung institute, UNITED KINGDOM

## Abstract

**Rationale:**

Respiratory syncytial virus (RSV) infection in preterm and newborn infants can result in severe bronchiolitis and hospitalization. The lamb lung has several key features conducive to modeling RSV infection in human infants, including susceptibility to human strains of RSV such as the A2, Long, and Memphis Strain 37 (M37). In this study, the kinetics of M37 infection was investigated in newborn lambs in order to better define clinical, viral, physiological, and immunological parameters as well as the pathology and lesions.

**Methods:**

Newborn lambs were nebulized with M37 hRSV (6 mL of 1.27 x 10^7^ FFU/mL), monitored daily for clinical responses, and respiratory tissues were collected from groups of lambs at days 1, 3, 4, 6, and 8 post-inoculation for the assessment of viral replication parameters, lesions and also cellular, immunologic and inflammatory responses.

**Results:**

Lambs had increased expiratory effort (forced expiration) at days 4, 6, and 8 post-inoculation. Nasal wash lacked RSV titers at day 1, but titers were present at low levels at days 3 (peak), 4, and 8. Viral titers in bronchoalveolar lavage fluid (BALF) reached a plateau at day 3 (4.6 Log_10_ FFU/mL), which was maintained until day 6 (4.83 Log_10_ FFU/mL), and were markedly reduced or absent at day 8. Viral RNA levels (detected by RT-qPCR) in BALF were indistinguishable at days 3 (6.22 ± 0.08 Log_10_ M37 RNA copies/mL; mean ± se) and 4 (6.20 ± 0.16 Log_10_ M37 RNA copies/mL; mean ± se) and increased slightly on day 6 (7.15 ± 0.2 Log_10_ M37 RNA copies/mL; mean ± se). Viral antigen in lung tissue as detected by immunohistochemistry was not seen at day 1, was present at days 3 and 4 before reaching a peak by day 6, and was markedly reduced by day 8. Viral antigen was mainly present in airways (bronchi, bronchioles) at day 3 and was increasingly present in alveolar cells at days 4 and 6, with reduction at day 8. Histopathologic lesions such as bronchitis/bronchiolitis, epithelial necrosis and hyperplasia, peribronchial lymphocyte infiltration, and syncytial cells, were consistent with those described previously for lambs and infants.

**Conclusion:**

This work demonstrates that M37 hRSV replication in the lower airways of newborn lambs is robust with peak replication on day 3 and sustained until day 6. These findings, along with the similarities of lamb lung to those of infants in terms of alveolar development, airway branching and epithelium, susceptibility to human RSV strains, lesion characteristics (bronchiolitis), lung size, clinical parameters, and immunity, further establish the neonatal lamb as a model with key features that mimic RSV infection in infants.

## Introduction

Human Respiratory Syncytial Virus (hRSV) is an enveloped, non-segmented, single stranded negative sense RNA pneumovirus of the paramyxoviridae family that causes lower airway respiratory disease in preterm newborns, term newborns, and elderly adults [1, 2]. It is the most important viral pathogen causing acute lower respiratory tract infections (ALRI) in infants younger than 5 years old and it is estimated to have resulted in ~3.4 million hospitalizations and ~200,000 deaths worldwide in 2005 [3]. RSV is transmitted by direct and indirect contact of nasal or oral secretions from an infected individual and primarily targets the lower airway respiratory epithelium (bronchioles) [4]. Clinical signs in infants and in children develop four to six days after infection with RSV, and usually subside after one to two weeks [5]. These signs vary with severity of disease and range from mild flu-like symptoms (coughing, sneezing, fever, and loss of appetite) in 25% to 40% of first-time exposed infants to severe bronchiolitis with or without pneumonia (rapid breathing, difficulty breathing, and wheezing) necessitating hospitalization in 0.5% to 2% of infants[6]. In very young infants, irritability, decreased activity, and apnea may be the only symptoms of infection. These clinical symptoms have been attributed to both the immune response to RSV, as well as the direct damage to RSV-infected bronchiolar epithelium [7, 8].

Current treatment of RSV infection is limited to supportive care. There exists an inhaled nucleoside analog (Ribavirin) that is approved for therapeutic use but which has limited treatment efficacy, as well as a monoclonal antibody (Synagis^®^, palivizumab), but whose use is limited to prophylactic application in high risk infants [9]. Two major hurdles in the development of preventative and therapeutic regimens are (i) the safety considerations following vaccination in young infants exemplified by the disastrous initial formalin-inactivated vaccine clinical trials where vaccination potentiated the disease rather than being protective and (ii) the lack of an available, clinically relevant model of RSV infection [10].

Animal models developed to study RSV infection include mice, cotton rats, ferrets, non-human primates, cattle, and lambs [9]. Lambs have several biological features that closely mimic RSV infection in human infants such as: similarities to human infants in lung development, lung structure and airway branching, cellular composition and immune responses, survival after late-preterm birth, susceptibility to various strains of RSV including human strains (Long, A2, and Memphis Strain 37), similar histologic lesions and lung size to human infants, and the ability to obtain lambs lacking maternal antibodies [11–15]. Despite these advantages and the many previous and on-going studies of RSV infection in lambs, the progressive development of clinical signs, lung pathology and inflammatory/immune responses over time after inoculation with a human strain of RSV have not been fully characterized. Thus, the aim of this study was to gain further insight and understanding of the effects of RSV infection in the neonatal lamb model throughout infection (days 1, 3, 4, 6 and 8 after inoculation) with the human RSV Memphis Strain 37. This kinetic information is needed in order to more fully characterize and utilize the lamb model for therapeutic and vaccine studies.

## Material and Methods

### Experimental design

Colostrum-deprived neonatal lambs (2–7 days of age) received daily antibiotics (Ceftiofur, Pfizer, New York, NY; 1–2 mg/kg, intramuscular) to prevent secondary bacterial infections. They were randomly assigned to five M37 hRSV-infected groups, of 3 lambs (n = 3). Each lamb received three 2-mL installments of 1.27 x 10^7^ FFU/mL in DMEM over a 23-minute period using a PARI LC Sprint^™^ nebulizer (PARI Respiratory Equipment, Inc., Lancaster, PA, USA) at 4L/min at 16 PSI (Philips Respironics Air Compressor, Andover, MA, USA) attached to a conical mask fitted with a round rubber diaphragm with a pre-cut center hole through which the nose and mouth of the lamb was inserted (MidWest Veterinary Supply, Inc., Burnsville, MN). Following infection, lambs were euthanized by sodium pentobarbital overdose and necropsied at different days post viral infection (p.i.): days 1, 3, 4, 6, and 8 p.i. After euthanasia the thorax was opened, lungs were removed, and RSV gross lesions (not including bacterial pneumonia lesions) were scored and photographed *ex vivo*. Tissue samples were collected from each lung lobe of all animals in the same manner, with uniform sampling of each lobe, and avoiding areas of bacterial pneumonia. Before lung dissection, bronchoalveolar lavage fluid (BALF) was collected from the right caudal lung lobe for infectious Focus-Forming Unit (FFU) assay and RT-qPCR for M37 hRSV total nucleoprotein RNA and accessory lobe, for cytology (total and differential cell counts), as described below. Three samples from each lobe were snap frozen in liquid nitrogen for reverse transcription quantitative polymerase chain reaction (RT-qPCR), and two samples from each lobe were placed in tissue cassettes and put in 10% neutral-buffered formalin for histological and immunohistochemical analyses.

Due to limitations in housing and number of lambs that can be handled in one study, the day 6 assessments were derived from an additional group of 3 lambs that was infected shortly after the necropsy of the other groups. The procedures, viral stock used and animal handling were identical to the viral kinetic (VK) study. The only difference was that the day 6 animals were slightly younger and lighter than the VK study animals as these came from another supplier (mean bodyweights of 3.4 ± 0.17 vs 6.3 ± 0.24 for the VK study on day 0; mean± se). In accordance to the 3R-principles, control lambs were not included in this study as previous work in our lab showed that non-infected lambs entirely lacked evidence of clinical illness, lung pathology, or immune and inflammatory changes that are consistent with M37 hRSV infection [1, 16–20]. Animal use and experimental procedures were approved by Iowa State University’s Animal Care and Use Committee (IACUC).

### Virus

Memphis 37 (M37) RSV is a wild type RSV-A, first isolated from a 4 month old infant [21] and used in human clinical studies [22–24]. The Memphis 37 RSV strain used in this study was passaged 6 times on Vero cells then twice on HEp-2 cells. Sucrose was added to 20% and the virus stock was frozen at −80°C and titered for infectivity on HEp-2 cells as we have characterized previously in this model [20].

### Monitoring of clinical signs

Lambs were monitored daily for body weights, rectal temperatures, heart rate and percent blood oxygenation measurements (PalmSAT^®^ 2500A VET pulse oximeter, Nonin Medical Inc., Plymouth, MN, USA), and manual heart and respiratory rates (by auscultation). Increased expiratory effort (forced expiration) was scored daily as were animal “wheeze” scores (Table 1).

**Table 1:** Scoring criteria for lung function by auscultation.

Score	Expiratory efforts	Wheezing (High-pitched whistling sound made while breathing)
0	No expiratory effort	No wheeze
1	Earliest detection of increased expiratory effort	Earliest detectable wheeze by auscultation
2	Moderate expiratory effort (>1sec) observed with some abdominal effort	Audible wheeze in all lung fields by auscultation
3	Expiratory effort (>3 sec) with hard abdominal effort	Wheeze audible without stethoscope

### Collection of nasal wash fluid (NWF)

Just before euthanasia the nasal cavity of each lamb was washed with double-modified Iscove’s medium (DMIM) containing 42.5% Iscove's modified Dulbecco's medium, 7.5% glycerol, 1% heat-inactivated FBS, 49% Dulbecco’s Modified Eagle medium (DMEM), and 5 μg/mL kanamycin sulfate. Using a 6-mL syringe fitted with a mucosal atomization device (MAD) conical foam end-piece (Intranasal Mucosal Atomization Device, Wolfe Tory Medical, Inc., Salt Lake City, UT, USA) a single 5 mL aliquot of DMIM was instilled into the right nare, and then (while still preserving a good seal between the nare and the MAD-device conical end-piece), any out-coming fluid was extracted fairly quickly, in one motion, back into the delivery syringe to collect (1.3–2.5 mL) NWF; which was dispensed into a 15 mL conical tube and placed on ice.

### Collection of bronchoalveolar lavage fluid (BALF)

Following euthanasia the lungs of each lamb were removed and each left and right lung was separated and weighed. The excised right caudal lung lobe was instilled with 5 mL of cold DMIM (42.5% Iscove's modified Dulbecco's medium, 7.5% glycerol, 1% heat-inactivated FBS, 49% DMEM, and 5 μg/ml kanamycin sulfate) after which 1 mL of the resulting BAL fluid was placed on ice and spun down for 5 minutes in a centrifuge at 3,000 x g to pellet large debris. Approximately 800–850 μL of each supernatant was collected and then spun through 850 μL-capacity 0.45 μm Costar SPIN-X filter (microcentrifuge 15,600 x g) for 5 minutes before being used in the standard infectious focus forming unit assay (FFU).

### Gross lesions evaluation and scoring

Following euthanasia, the thorax was opened and the heart and esophagus were removed from the lungs. The percentage parenchymal involvement of gross RSV lesions was scored for each individual lung lobe, and if present, the area and amount of lung lobes affected by bacterial pneumonia was also recorded. The percentage of a specific lobe tissue that was affected by RSV in relation to the overall lobe tissue being scored was estimated based on a score as done previously [1]. Mean percentage averages per lobe were calculated for each day of necropsy.

### Histologic evaluation and scoring

A histologic score was given by determining percent involvement followed by conversion to an additional integer-based consolidation scale used by our laboratory previously [1] wherein: 0% consolidation = 0; 1%-9% consolidation = 1; 10%-39% consolidation = 2; 40%-69% consolidation = 3; 70%-100% consolidation = 4. In total, multiple fields from 4 slides per animal were scored for the lesions. Each slide contained 2 different sections from the same lobe. Histologic score for each animal was the mean of all 4 slides and group averages were calculated for the alveolar consolidation score. In addition to the consolidation score, bronchitis/bronchiolitis, neutrophil infiltration, peribronchiolar and perivascular infiltration of lymphocytes, syncytial cell formation, and epithelial alterations were also scored according to criteria indicated in Table 2.

**Table 2:** Histologic lung lesion scoring criteria.

Score	Bronchitis	Bronchiolitis	Syncytial cells	Epithelial necrosis (bronchi or bronchioles)	Epithelial hyperplasia (bronchi, bronchioles)	Neutrophil (in bronchi, bronchioles or alveoli)	Bi nodules (these are peribronchiolar lymphocytic infiltrates)	Vessel nodules (lymphocytic infiltrates around blood vessels)	Eosinophilic infiltrates in bronchi or bronchioles, alveoli	Eosinophilic infiltrates in vessels	Eosinophils in vessel lumens
0	no remarkable lesions	no remarkable lesions	none	none	none	none	none	none	none	none	none
1	minimal detectable lymphoplasmacytic infiltrates in lamina propria and adventitia	minimal detectable lesion (epithelial degeneration) in one or a few bronchioles per 20x field	one distinct syncytial cell	minimally detectable in one or a few per 20x field	minimally detectable in one or a few airways per 20x field	minimally detectable	earliest detectable lymphocytic infiltrates in the adventitia	earliest detectable lymphocytic infiltrates in the adventitia	minimally detectable	minimally detectable	minimally detectable
2	segmental to circumferential infiltrates	epithelial degeneration involving less than 10% of the airway lumen; minimal neutrophils, cell debris; adventitial lymphocytes in multiple bronchioles	up to three in three 20x fields	10% in multiple airways per 20x field	10% of airway per 20x field	10 or less neutrophils in one or a few airways/alveoli	segmental to circumferential infiltrates	segmental to circumferential infiltrates	up to five eosinophils in one to several airways/alveoli per 20x field	up to five eosinophils associated with a vessel	up to five in one or a few vessels
3	dense infiltrates	epithelial degeneration involving >10–50% of the airway lumen with cell debris, neutrophils; adventitial lymphocytes; multiple bronchioles	more than three in three 20x fields	10–50% in multiple airways per 20x field	10–50% in multiple airways per 20x field	10 or more neutrophils in several airways/alveoli	circumferential infiltrates that expand more than three cells wide	circumferential infiltrates that expand more than three cells wide	over five in over five airways per 20x field	> five in over five vessels per 20x field	> five in over five vessels
4	infiltrates with nodular aggregates	circumferential bronchiolitis with dense adventitial lymphocytes; multiple bronchioles	Numerous in three 20x fields	circumferential in multiple airways	circumferential	10 or more involving many/most airways/alveoli	circumferential infiltrates that form nodules	circumferential infiltrates with nodules	dense accumulations of eosinophils	dense accumulations of eosinophils	dense accumulations

### Immunohistochemistry for viral antigen detection

Immunohistochemistry for the detection of RSV antigen was performed on 5 μm-thick formalin-fixed paraffin-embedded (FFPE) lamb lung tissue sections taken from the right and left cranial, left middle, and left caudal lung lobes of each animal in accordance with methods published previously [17, 25], but with the following variations: instead of Pronase E antigen retrieval, heated buffer antigen retrieval was performed in TRIS-EDTA-0.05% Tween 20, pH 9.0 in a pressure cooking device (Decloaking Chamber^™^ Plus, Biocare Medical, Concord, CA) using the factory default 40-minute program (125°C reached in 18 minutes and cooling to 80°C in another 22 minutes). Primary polyclonal goat anti-RSV (all antigens) antibody (EMD Millipore Corporation, Billerica, MA, USA) was applied for 90 minutes at room temperature diluted 1:500 in TBS-tween containing 10% NSS and 3% BSA. After rinsing with TBS-tween, biotinylated rabbit anti-goat secondary antibody (Kirkegaard-Perry Labs, Gaithersburg, MD, USA) diluted 1:300 in TBS-tween containing 10% NSS and 3% BSA was applied for 45 minutes, after which slides were rinsed with TBS-tween, treated with 3% H_2_O_2_ in TBS-tween for 25 minutes, rinsed and then incubated with streptavidin-conjugated HRP (Invitrogen) diluted 1:200 in TBS-tween for 30 minutes. Development of the color was performed in custom 12-slide plastic containers (Antibody Amplifier^™^ containers, ProHisto, LLC, Columbia, SC, USA) by applying Nova Red (Vector Laboratories, Inc.) for about 90 seconds followed by copious rinses with ddH_2_O, counterstaining with Harris’ hematoxylin (for 2 minutes), bluing with alkaline Scott’s water (for 1 minute), dehydration and coverslipping with Permount mounting medium (Sigma, St. Louis, MO, USA). 20 unique 10X fields on each slide (containing two lung sections each) were assessed for RSV antigen staining by counting positively-stained cells within bronchioles and alveoli. The mean number of stained bronchi/bronchioles and alveoli per field were counted for each day of necropsy.

### Reverse transcription quantitative polymerase chain reaction (RT-qPCR) assessment of RSV and chemokine gene mRNA expression levels in lamb lung

For each animal, tissue samples from right and left cranial, left middle and left caudal lung lobes (0.3–0.4 g of each lobe) were homogenized for total RNA isolation in TRIzol (Invitrogen) and previously described methods [1]. RNA was assessed for quantity and purity by spectrometry (Beckmann DU^®^ 640B, Beckmann Coulter Inc., Brea, CA, USA) and all OD_260nm/280nm_ values measured between 1.96 and 2.12. Agilent Bioanalyzer 2100 (Agilent Technologies, Santa Clara, CA, USA) analysis of RNA prior to DNase treatment consistently yielded RIN values ≥8.0 for all lamb lung RNA samples isolated this way [16]. Reverse transcription quantitative polymerase chain reaction (RT-qPCR) was performed using One-Step Fast qRT-PCR Kit master mix (Quanta, BioScience, Gaithersburg, MD, USA) in a GeneAmp 5700 Sequence Detection System (Applied Biosystems, Carlsbad, CA, USA) and PREXCEL-Q for all set up calculations [26, 27]. Primer and probe sequences for all targets were designed with ABI Primer Express 2.0, and have been used previously [1, 17, 28]. Primers and hydrolysis probe for targeting M37 hRSV NP RNA were designed using ABI Primer Express version 2.0 based on RSV accession number M74568. Thermocycling conditions were 5 minutes at 50°C; 30 seconds at 95°C; and 45 cycles of 3 seconds at 95°C and 30 seconds at 60°C. Samples and standards were assessed in duplicate, and each target gene quantification cycle (Cq) value was converted to a relative quantity (Q_r_) based on each target’s standard curve using: Q_r_ = E_AMP_^(b-Cq)^, wherein “b” and “E_AMP_” are the y-intercept and exponential PCR amplification value, respectively. E_AMP_ values were obtained from the slope (m) of each target standard curve by: E_AMP_ = 10^(-1/m)^, and all Q_r_ values interpolated from standard curves were normalized to total lung RNA per RT-qPCR (0.784 ng RNA/μL for all reactions). No-RT control (NRC) reactions gave either no signal or generated Cq values greater than 13 cycles later than those in the corresponding RT-qPCR target reactions.

### Reverse transcription quantitative polymerase chain reaction (RT-qPCR) for RSV in nasal wash fluid and bronchoalveolar lavage fluid

Viral RNA was quantified by reverse transcription quantitative polymerase chain reaction (RT-qPCR) in NWF and BALF obtained from each animal at necropsy. NWF was obtained from the right nasal cavity and BALF from the right caudal lung lobe of each animal (see section on NWF and BALF collection). Briefly, 100 μL of each collected fluid sample was pipetted directly into 1 mL of TRIzol (Invitrogen/Life Technologies, Carlsbad, CA, USA) on ice, inverted to mix, and then transferred to -80°C for storage until RNA isolation and subsequent RT-qPCR. Upon thawing, each 1.1 mL sample was vortexed for 10 seconds and allowed to sit at room temperature for 10 minutes. RNA isolation from NWF and BALF samples continued as per manufacturer’s instructions. The resulting (non-visible) RNA pellets, were each dissolved in 100 μL of nuclease-free water (Invitrogen/Life Technologies), vortexed thoroughly, microfuged briefly, warmed to 60°C for 3 minutes, vortexed for 5 seconds, microfuged briefly, then 80 μL of each was diluted 1:10 with a combination of 10 μL RNaseOUT^™^ (to 0.5 Units/ μL), and 710 μL nuclease-free water, then stored at 4°C prior to RT-qPCR. RT-qPCR for RSV was then carried out as described above in the section: “Reverse transcription quantitative polymerase chain reaction (RT-qPCR) assessment of RSV and chemokine gene mRNA expression levels in lamb lung”.

### Focus-forming unit (FFU) assay

Viral titers in both nasal wash fluid (NWF) and bronchoalveolar lavage fluid (BALF) from the right lung caudal lobe were determined using an infectious focus assay (FFU). In brief, 200 μL of serially-diluted NWF or BALF samples were applied to HEp-2 cells grown to 70% confluence in 12-well culture plates (Fisher Scientific, Hanover Park, IL) in DMEM media (Mediatech, Inc., Manassas, VA) supplemented to 10% with heat-inactivated fetal bovine serum (FBS) (Atlanta Biologicals, Atlanta, GA) and 50 μg/mL kanamycin sulfate (Invitrogen/Life Technologies). Each sample was analyzed undiluted and at four additional serial-dilutions of 1:10, 1:100, 1:1,000 and 1:10,000 in duplicate. Following a 48-hour incubation at 37°C, 5% CO_2_, the cells were fixed with cold 60% acetone/40% methanol solution for 1 minute. Overnight primary polyclonal goat anti-RSV (all antigens) antibody (EMD Millipore Corporation, Billerica, MA, USA) incubation was followed by washing and secondary antibody (Alexa Fluor^®^ 488 F(ab’)2 fragment of rabbit anti-goat IgG (H+L), Molecular Probes/Life Technologies) incubation for 30 minutes. Plates were rinsed and inspected for the presence of fluorescing foci of infection using the FITC/GFP filter on an inverted fluorescence microscope (Olympus CKX41, Center Valley, PA). Five or more fluorescing cells were counted as single focal events. An average of 40 counts in a 1:10-diluted (duplicate) sample indicated an original NWF or BALF sample “titer” of 2,000 [40 counts x dilution of 10 x 1,000 μL/mL]/200 μL assessed = 2,000 infectious focus-forming units/mL (FFU/mL).

## Results

### Clinical findings

**Fig 1:**
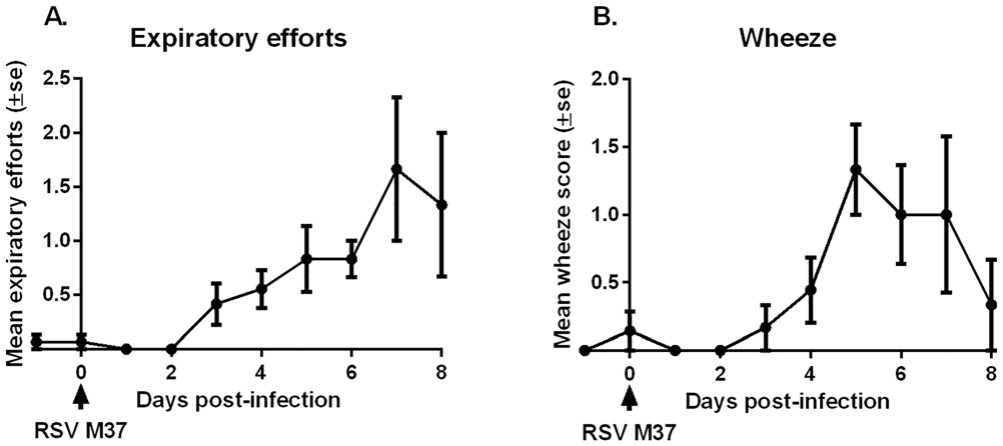
Respiratory distress score of lambs inoculated with human respiratory syncytial virus (hRSV) strain Memphis 37 (M37). Respiratory distress score was assessed daily for each lamb by auscultation or visual observation, and was categorized by expiratory effort **(A)** and wheezing **(B)**. Results are shown as mean ± standard error.

Following RSV-infection, there were no differences in weight gain, body temperature, heart rate, and percent blood oxygen saturation when compared to control lambs from previous studies in which the procedures, facilities, animal handling, and source and age of lambs were similar [1, 16–20]. Despite a small drop in mean blood oxygen saturation levels on day 6 (92.4% ± 2%; mean ± SD) when respiratory distress was present in most lambs, these remained above the 90% limit. Respiratory rates (not shown) were variable and non-predictable for each day of the study and were likely confounded by the heavy sampling schedule and the resultant stress level of the newborn lambs. Increased expiratory efforts and wheezing were the only noteworthy clinical features observed in RSV-infected lambs in this study. Following inoculation with M37 hRSV, expiratory efforts became apparent on day 3 in 4 out of 12 lambs (33%). On day 5 this proportion increased to 4 out of 6 lambs (66%) and on day 6 to 5 out of 6 lambs (83%). By day 7 all lambs (100%) had increased expiratory efforts, but on day 8 this proportion dropped to 2 lambs out of 3 (66%). The severity of expiratory efforts increased from day 3 to day 7 as shown by the mean expiratory effort score (Fig 1A). Similarly, wheezing was apparent on day 3 although only in 1 out of 12 lambs (8.3%). The proportion of lambs that developed wheeze gradually increased to reach a peak on day 5 (83.3%) and decreased on day 8 (33%). The mean wheeze score followed a similar time profile (Fig 1B).

### Gross and microscopic lung lesions

**Fig 2:**
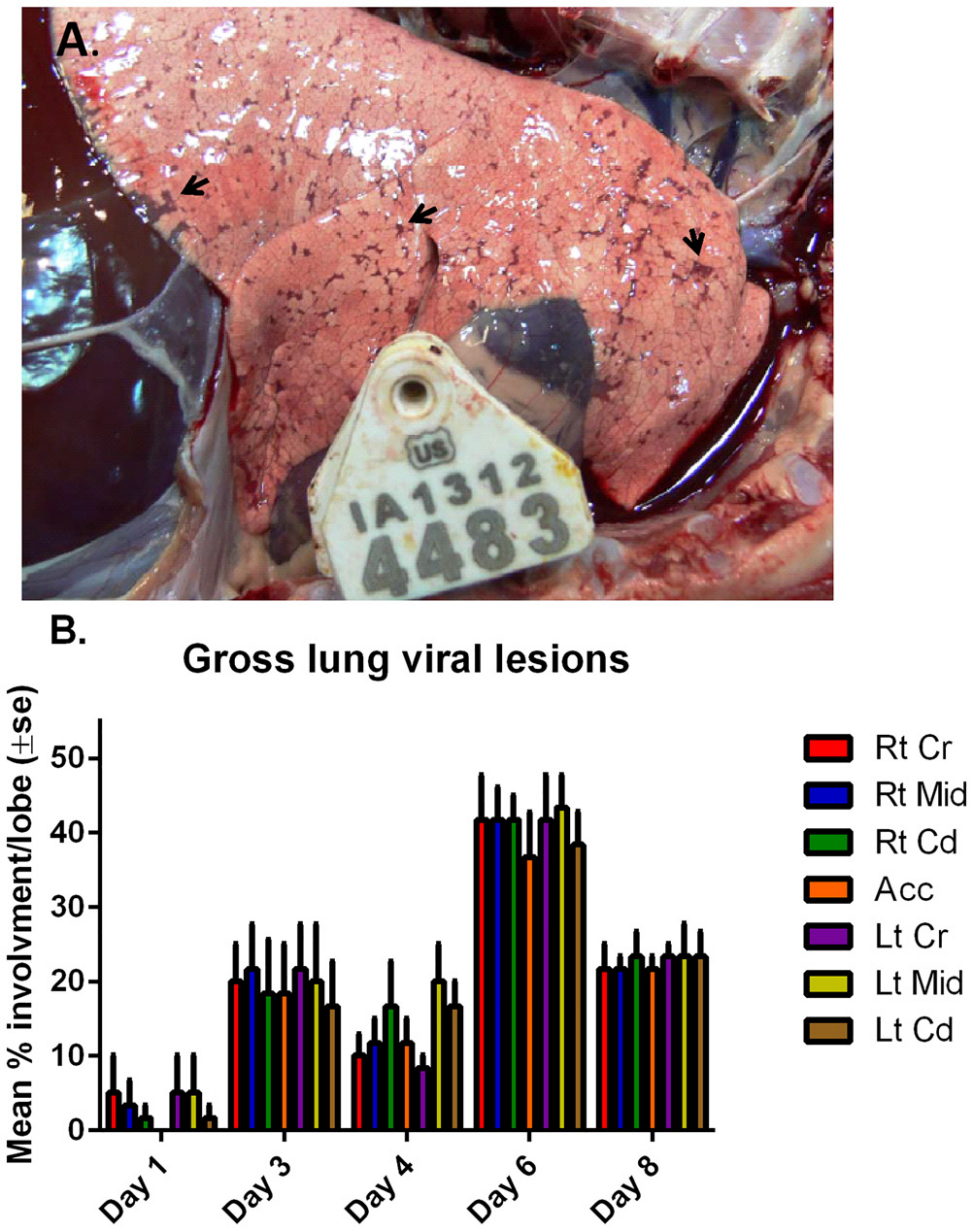
Viral gross lesions caused by M37 hRSV infection in neonatal lambs. **(A)** Picture of a lung on day 6 post-infection. Dark plum-red, well-demarcated foci of pulmonary consolidation are indicated by arrowheads. **(B)** Percentage parenchymal involvement was estimated for each lung lobe and mean percentage averages per lobe were calculated for each day of necropsy (± standard error). Legend: Rt Cr = Right cranial lobe; Rt Mid = Right Middle lobe; Rt Cd = Right Caudal lobe; Acc = Accessory lobe; Lt Cr = Left Cranial lobe; Lt Mid = Left Middle lobe; Lt Cd = Left Caudal lobe.

Following necropsy, gross examination of the lungs determined the percent of each lobe that was covered with typical RSV-induced lesions. In some cases, areas of lung with lesions suggestive of bacterial pneumonia were also present along with RSV-induced lesions, and lung lobes with lesions suggestive of bacterial pneumonia were recorded, but not scored as RSV lesions. RSV lesions were bilateral, evenly-distributed and characterized by multifocal to locally extensive dark plum-red, well-demarcated foci of pulmonary consolidation which varied from mild to severe (Fig 2A); consistent with RSV infection in lambs as reported previously [1, 17, 29]. In contrast to RSV lesions, lung lobes affected by bacterial pneumonias were mild, unilateral, and cranial ventral (right and middle lung lobes being mostly affected), and were characterized by multifocal, locally extensive, firm, red-brown areas. Following RSV-infection, gross RSV viral lesions were already detectable on day 1, and further increased by day 3, to reach maximal levels by day 6 of around 40% and decreased thereafter (Fig 2B). For the lambs used in this study, only the last group of lambs (10 and 12) necropsied at day 8, had gross lesions of bacterial pneumonia affecting the right middle lung lobe. For day 6 lambs, lamb 14 had bacterial pneumonia affecting the right cranial and middle lung lobes. Again, these lung regions bearing non-RSV-induced lesions were not sampled for attaining experimental endpoints.

**Fig 3:**
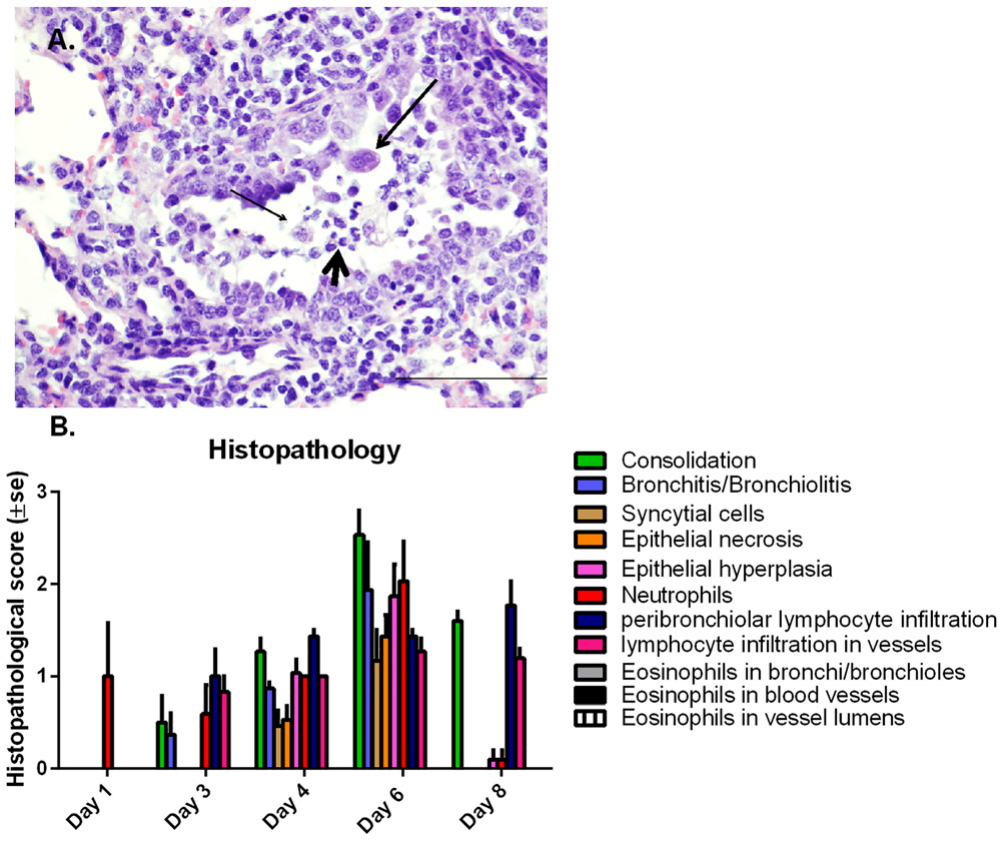
Microscopic lung lesions severity score in M37 hRSV infected neonatal lambs. **(A)** Histopathologic lesions included bronchiolitis with degenerate/necrotic individual epithelial cells (thin arrow), occasional syncytial cells (long arrow), accumulation of degenerate neutrophils (short arrow), and occasional macrophages. H&E Bar = 50 μm. **(B)** A histologic score was given by determining percent consolidation followed by conversion to an integer-based consolidation scale used by our laboratory previously [1]: 0% consolidation = 0; 1%-9% consolidation = 1; 10%-39% consolidation = 2; 40%-69% consolidation = 3; 70%-100% consolidation = 4. Group averages were calculated for alveolar and bronchiolar consolidation scores. In addition to the consolidation score, bronchitis, bronchiolitis, neutrophil infiltration, peribronchiolar and perivascular infiltration of lymphocytes, syncytial cell formation, and epithelial alterations were also scored. Results are indicated as mean ± standard error for each scored parameter.

Microscopically, lungs of infected lambs had multifocal to coalescent foci of an inflammatory infiltrate that partially filled the lumen of bronchi/bronchioles, alveolar spaces, and alveolar septa. The airway lumen was also partially occluded by seroproteinaceous fluid and cell debris intermixed with mucin. These lesions increased progressively with time and were similar to those described previously with M37 hRSV and with hRSV A2 strains [1, 17, 18, 25]. On day 1 p.i., small numbers of neutrophils were present within the lumen of bronchioles and occasional bronchi. On day 3, microscopic lung lesions were characterized by mild to moderate infiltrates of neutrophils in bronchiolar lumens with small amounts of seroproteinaceous fluid and mucin. A mild but detectable infiltration of lymphocytes in the tunica adventitia of bronchioles and nearby blood vessels was also present. There was degeneration (cells with rounded cell borders and basophilic/pyknotic nuclei) of epithelial cells in bronchioles (Fig 3A). The intensity of the lesions were further increased on day 4 and characterized by the neutrophil infiltration with sloughed, necrotic epithelial cells, seroproteineous fluid and small amounts of mucin in bronchioles and bronchi along with occasional macrophages and the formation of occasional syncytial cells in bronchio-alveolar spaces. The alveolar septa were mildly to moderately thickened by hyperplasia of type II pneumocytes and the bronchioles were surrounded by moderate to mild numbers of lymphocytes and plasma cells; a few lymphocytes were present within the alveolar septa. By day 6, all observed lesions present on day 4 peaked, with the notable exception of lymphocytic infiltration in the peribronchiolar region and blood vessels. Neutrophils were prominent on day 6 p.i. but reduced/absent on day 8 p.i. which is consistent with our previous studies in the lamb model [17, 28]. Peribronchiolar and perivascular lymphocyte infiltration was increased on day 8 while all other parameters were reduced (Fig 3B).

### Viral titers, viral RNA levels and viral antigen expression

Levels of M37 hRSV total nucleoprotein RNA in lung tissue, BALF, and NWF were measured by RT-qPCR, while cultivatable virus was quantified by infectious focus (FFU) assay in NWF and BALF collected on each day of necropsy. Viral titers and viral RNA in BALF and lung tissue increased progressively from day 1 to day 3 and were sustained or increased slightly until day 6. On day 8, viral titers and RNA decreased substantially as observed for all other virology endpoints (viral antigen expression, gross lung lesions, and microscopic lesions). In contrast, viral titers and viral RNA levels in NWF were more variable when comparing them to levels in BALF and lung tissue. Viral titers in NWF were highest at day 3 (1.7 log_10_ FFU/mL), whereas this was the case on day 6 (2.9 log_10_ RNA copies/mL) for viral RNA. Overall, intranasal viral replication was substantially lower than viral replication in the lung (Table 3) and may be due to the administration of virus by nebulization which may bypass the nose to some extent or be indicative of a lower permissiveness of lamb nasal epithelial cells for RSV replication.

**Table 3:** Quantification of RSV replication via RT-qPCR and infectious focus assay in lambs inoculated with M37 hRSV.

	Viral load (Nasal Washes)*		Viral load (BALF)*		Viral transcripts(Lung tissue)
	Viral culture (Log_10_ FFU/mL ± se)	RT-qPCR (Log_10_ M37 RNA copies/mL ± se)	Viral culture (Log_10_ FFU/mL ± se)	RT-qPCR (Log_10_ M37 RNA copies/mL ± se)	RT-qPCR (Log_10_ M37 RNA copies/mg lung tissue ± se)
**Day 1**	BDL (0.7)	1.44 ± 1.1	2.53 ± 0.09	3.80 ± 0.03	4.81 ± 0.15
**Day 3**	1.7 ± 0.4	1.68 ± 1.4	4.60 ± 0.32	6.22 ± 0.08	6.51 ± 0.08
**Day 4**	0.6 ± 0.1	0.42 ± 0.1	3.94 ± 0.25	6.20 ± 0.16	6.67 ± 0.25
**Day 6**	0.99 ± 0.3	2.89 ± 0.34	4.83 ± 0.04	7.15 ± 0.2	7.63 ± 0.07
**Day 8**	0.8 ± 0.1	1.46 ± 1.2	1.02 ± 0.32	4.70 ± 0.38	5.24 ± 0.26

*BDL = below detection limit. All samples BDL were assigned a value of 0.7 log_10_ FFU/mL for culture and 0.3 log_10_ RNA copies/mL for RT-qPCR. Values in brackets depict the standard error.

**Fig 4:**
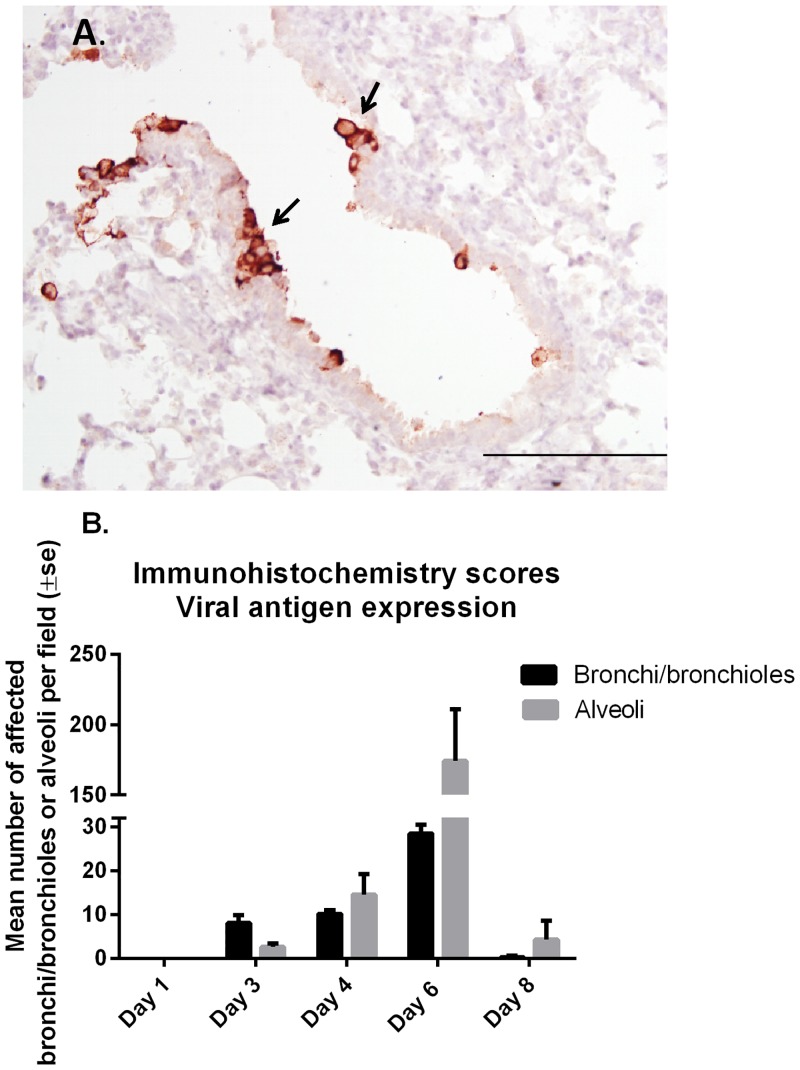
Immunohistochemistry and scoring of RSV antigen expression in lambs inoculated with M37 hRSV. Immunohistochemistry was used to detect viral antigen using an all-antigens polyclonal antibody for RSV. **(A)** RSV immunoreactivity is shown within epithelial cells lining the bronchioles (brown cells). Bar = 50 μm **(B)** The mean number of virally-infected bronchi/bronchioles and alveoli per field was counted for each day of necropsy.

With immunohistochemistry, RSV antigen was present in areas with microscopic lesions. Within these areas, RSV antigen was present in the entire cytoplasm of epithelial cells lining bronchi and bronchioles, alveoli, and the cytoplasm of occasional macrophages (Fig 4A). On day 1 p.i. no viral antigen expression was detected in the lungs of infected lambs and was only apparent on day 3 predominantly in the epithelial cells of bronchi and bronchioles when compared to the alveoli. There was an increasing progression of viral antigen expression in both bronchi/bronchioles and alveoli, which reached a peak on day 6 with a marked shift in viral antigen expression occurring in the alveoli. Degenerate and necrotic epithelial cells within lumens of bronchioles also contained viral antigen at days 3, 4, and 6. On day 8, viral antigen expression in lung tissue had decreased substantially (Fig 4B) consistent with a decline in RSV titers in lung (Table 3).

### Chemokine and cytokine expression in lung tissue

**Fig 5:**
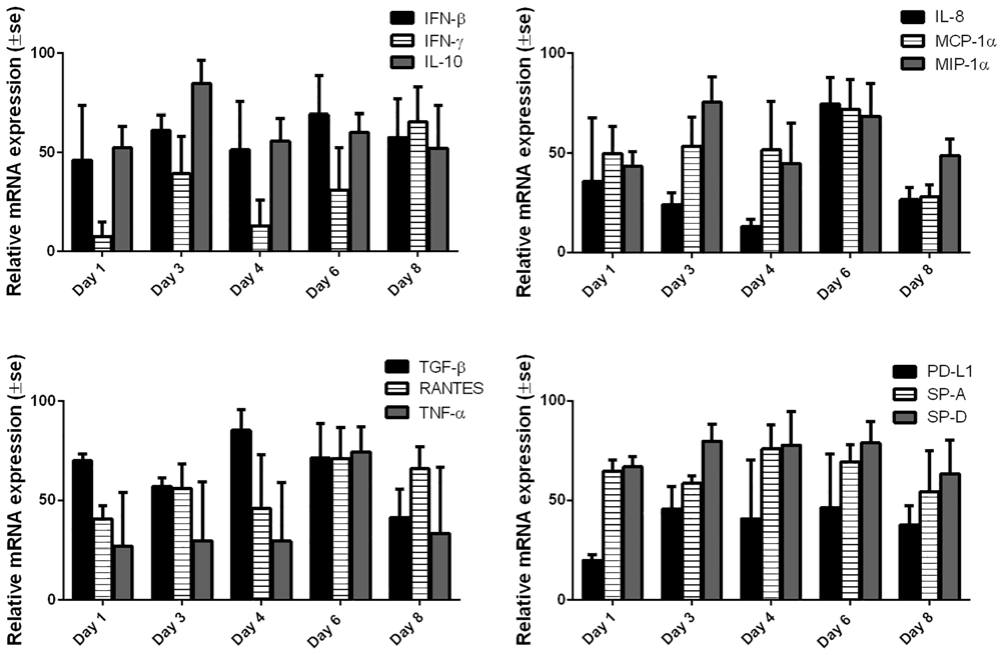
Lung chemokine mRNA expression assessed by RT-qPCR in lambs inoculated with M37 hRSV. Lung tissue obtained from each animal was evaluated for the following mRNA targets: surfactant protein A (SP-A), surfactant protein D (SP-D), interleukin 8 (IL-8), interleukin 10 (IL-10), macrophage inflammatory protein 1 alpha (MIP-1α), monocyte chemotactic protein 1 alpha (MCP-1α), tumor necrosis factor alpha (TNF-α), transforming growth factor beta (TGF-β), interferon beta (IFN-β), interferon gamma (IFN-γ), programmed cell death 1 ligand 1 (PD-L1), and regulated on activation normal T-cell expressed and secreted (RANTES). Mean relative mRNA expression was calculated for each target with respect to each day of necropsy. Relative mRNA expression means: relative to the total amount of RNA loaded per reaction (which is kept constant) and relative to the values established by the standard curves for each target.

Lung cytokine mRNA expression levels were quantified by RT-qPCR and demonstrated varying patterns of expression in M37 hRSV-inoculated lambs throughout infection. While IL-10 expression levels were highest on day 3 post-infection other chemokines and cytokines had maximal expression at later timepoints (e.g. TGF-β on day 4; IL-8, RANTES and MCP-1α on day 6 and IFN-γ on day 8) (Fig 5). Similarly, previous results from our laboratory have shown that MCP-1α, MIP-1α, RANTES, IFN-γ, and IL-8 were increased upon RSV-infection in neonatal lambs on day 6 [1], whereas the anti-inflammatory mediator, IL-10, was down regulated at day 6 post-infection, but increased on day 3 post-infection [28].

## Discussion

This study aimed to determine the time course of M37 hRSV replication in neonatal lambs and the corresponding pathophysiology i.e. clinical signs (wheezing, respiratory distress), airway inflammation, and lung histopathology. Such a time course is difficult to assess in infants since the diagnosis is often only done at the time of or near peak viral titers.

Colostrum-deprived neonatal lambs are highly relevant for the study of RSV infection and may serve as a model of RSV infection in human infants due to the natural susceptibility of lambs to ovine, bovine and human strains of RSV as well as similarities in disease pathogenesis and anatomical, physiological and developmental similarities to that of human infants [13–15]. Experimentally, it has been shown that lambs and other ruminants can be infected with human or bovine RSV strains and that the infection induces lung lesions that resemble those observed in human RSV pathology such as bronchiolitis with epithelial cell necrosis, syncytial cell formation, hyperplasia of nearby epithelium and infiltrates of neutrophils with occasional macrophages [1, 11, 17, 29–33]. Moreover, RSV infected lambs develop mild to moderate clinical symptoms such as expiratory effort, fever, tachypnea, wheeze, malaise and listlessness [1, 11, 17, 29, 32] and formalin-inactivated RSV vaccination in lambs induces enhanced lesions upon RSV infection [19] as observed in infants [34, 35]. To date, and to our knowledge, no study has yet specifically addressed the time course of viral replication, histopathology, airway inflammation, and associated clinical symptomatology (wheezing, respiratory distress) in neonatal lambs following infection by nebulized inhalation of the M37 hRSV strain. The purpose of this study, therefore, was to define the time course of Memphis 37 RSV replication in the lungs of neonatal lambs and the corresponding clinical features and pathophysiology.

Following RSV infection, this study demonstrates that there is robust viral replication as determined by titers and mRNA levels in the lungs, which peaked on day 3, were sustained until day 6 and decreased by day 8. Viral replication was present in the nasal cavity with maximal titers detected at days 3 (cultivatable virus) and 6 (mRNA) post inoculation, but this replication was less robust compared to lung, as viral titers were ~3 Log lower in the nasal wash fluid than in lung on day 3. Such differences in NWF and BALF titers may be related to the administration route which partly bypasses the nasal meatus and also because the sampling of nasal cavity (washes of a cavernous space) differs from solid lung tissue and BALF (taken from a bronchus directly at post mortem). The kinetics of RSV RNA expression levels in BALF and lung tissue displayed a similar profile. Viral antigen expression followed a similar time-profile as titers and mRNA levels, albeit with a delay, and was detected in intact and degenerate/necrotic bronchiolar epithelial cells and ciliated airway epithelial cells in bronchi; cell types that were shown to be permissive to RSV infection [36–41], as well as in the cytoplasm of occasional macrophages, consistent with the distribution of virus-infected cells in fatal cases of RSV-infection [33]. Viral antigen expression in bronchi/bronchioles and alveoli for each day post-inoculation followed the progression/severity of microscopic lung lesions, gross lung lesions, and respiratory distress.

Microscopically, the bronchiolar epithelia had evidence of necrosis on days 3 and 4 and epithelial hyperplasia, which increased until day 6. The infected bronchiolar cells can, as a result, become degenerate and necrotic and contribute to the cell debris entering and partially occluding the airway lumen. In addition, the necrotic areas and the inflammatory mediators released in this process facilitate neutrophil infiltration and accumulation of seroproteinaceous fluid and mucin, all of which can further occlude the airway lumen. Lymphocytes were first observed by day 3 and 4 and appeared to achieve peak levels by day 6 to 8. Immune cell infiltrates accumulated in the tunica adventitia of bronchi, bronchioles and small blood vessels, and slightly more macrophages were observed in the alveolar and bronchiolar lumens. On day 8 post-infection, airway lumens were observed to contain only occasional neutrophils and macrophages, while infiltrates of lymphocytes and plasma cells remained present in the adventitia, reflecting a change from neutrophilic to lymphocytic and plasmacytic inflammation. The role of lymphocytes in RSV clearance and convalescence has previously been evidenced in human and mouse studies [42–44] and the failure to develop an adaptive cytotoxic T lymphocyte response has been proposed to be related to the pathogenesis of RSV infection of the lower respiratory tract [45]. Conversely, neutrophils are the most abundant cell type recovered from the respiratory tract in infants-hospitalized for RSV disease [46–48] and it has been suggested that they can contribute significantly to epithelial cell damage and to disease severity [49, 50].

In addition to inflammatory cell influx in the lungs, it has been shown that chemokines and cytokines, such as IL-8, RANTES, MIP-1α, IL-6 and IL-10 are increased in RSV-infected infants [49, 51–57] and likely to promote the intense inflammatory process present in the airways of these infants. For this reason, we sought to investigate the time course of chemokine expression in lung tissue from RSV-infected lambs. Expression of proinflammatory chemokines and cytokines such as IL-8, IFN-γ, IFN-β, MCP-1α, MIP-1α and TNF-α or anti-inflammatory cytokines such as IL-10 was detected and was consistent with previously published data [1, 28]. Peak expression of IL-8 was present at day 6 post-infection and coincided with peak neutrophil influx in the lungs of RSV-infected lambs which indicates that the increased chemokine expression contributed to neutrophil infiltration into the site of intense RSV infection, in accordance with the role of IL-8 in neutrophil chemotaxis [7]. In contrast, IFN-γ expression, a cytokine mainly produced by NK cells and activated CD4^+^ and CD8^+^ T cells that promotes cell-mediated immune responses to intracellular pathogens, was maximal at day 8 post-infection at a time when peak lymphocyte lung infiltration was noted. Taken together, this data suggests that RSV infection in neonatal lambs results in an initial innate inflammatory response, the peak of which coincides to that of peak disease, and is characterised by IL-8 secretion and neutrophil influx. During the transition from the initial inflammatory response, there is infiltration of lymphocytes and, as the disease resolves, the inflammatory response is characterised by IFN-γ secretion and continued lymphocyte influx. Interestingly, a suppressed lymphocyte function and increased plasma IL-8 levels were shown to be markers of severe disease in RSV bronchiolitis [58].

The most interesting results, however, obtained in the current study were those related to the clinical parameters. Infants with RSV-disease typically develop clinical manifestations such as bronchiolitis and pneumonia symptoms of which include wheezing, crackles, rhonchi, tachypnea, nasal flaring, and intercostal muscle retractions [59, 60]. In our study, the neonatal lambs developed respiratory distress (forced expiration, abdominal breathing and wheeze) following RSV-infection consistent with previous reports [1, 18]. The progression of respiratory distress coincided with that of viral replication (titers, viral RNA and viral antigen expression), lung gross viral lesions, and histopathological changes. The resultant partial occlusion of the small airways by sloughed epithelial cells, inflammatory cells, respiratory secretions and mucous plugs as observed microscopically may lead to air trapping, lung hyperexpansion and increased airway resistance causing respiratory distress [61]. The deterioration of lung function and clinical symptomatology thus appears to be a direct effect of viral replication and its induction of bronchiolar/lung pathology. In experimentally-infected adults, a similar close temporal association between onset, peak, and clearance of viral replication, and the onset, peak, and resolution of the disease, has been described as well. However, viral replication and disease are limited to the upper airways in this human model [22].

This viral kinetic study of M37 hRSV in newborn lambs establishes a baseline of clinical features and pathology in a model where RSV infection and the consequences thereof resembles that of human infant RSV disease in the lower airways and may serve as a valuable tool to assess vaccine and antiviral drug safety and efficacy.
